# Gut microbiota interacts with intrinsic brain activity of patients with amnestic mild cognitive impairment

**DOI:** 10.1111/cns.13451

**Published:** 2020-09-15

**Authors:** Ping Liu, Xi‐Ze Jia, Yi Chen, Yang Yu, Kan Zhang, Ya‐Jie Lin, Bao‐Hong Wang, Guo‐Ping Peng

**Affiliations:** ^1^ Department of Neurology College of Medicine The First Affiliated Hospital Zhejiang University Hangzhou China; ^2^ Center for Cognition and Brain Disorders Hangzhou Normal University Hangzhou China; ^3^ Collaborative Innovation Center for Diagnosis and Treatment of Infectious Diseases State Key Laboratory for Diagnosis and Treatment of Infectious Diseases College of Medicine The First Affiliated Hospital Zhejiang University Hangzhou China

**Keywords:** amnestic mild cognitive impairment, gut microbiota, interaction, resting‐state functional magnetic resonance imaging

## Abstract

**Aims:**

To explore the potential relationships among gut microbiota (GM), local brain spontaneous activity, and neuropsychological characteristics in amnestic mild cognitive impairment (aMCI) patients.

**Methods:**

Twenty aMCI and 22 healthy control (HC) subjects were recruited. The GM composition was determined by 16S ribosomal RNA gene sequencing. Resting‐state functional magnetic resonance imaging scans were performed, and fractional amplitude of low‐frequency fluctuations (fALFF) was calculated across different frequencies. The Spearman or Pearson correlation analysis was used to analyze the relationship between spontaneous brain activity and cognitive function, and GM composition.

**Results:**

aMCI patients had altered GM state and local spontaneous brain activity as compared with HC subjects. Correlation analysis showed that aMCI and HC groups had different “GM‐intrinsic brain activity interaction” patterns. In aMCI group, at the typical band (0.01‐0.08 Hz), the relative abundance (RA) of Bacteroides from phylum to genus level was negatively correlated with fALFF value of cerebellar vermis IV‐V, and the *Ruminococcaceae* RA was negatively correlated with fALFF values of left lenticular nucleus and pallidum. The *Clostridiaceae* RA and *Blautia* RA were positively correlated with the left cerebellum lobules IV‐V at the slow‐4 band (0.027‐0.073 Hz). The *Veillonellaceae* RA was positively correlated with fALFF values of left precentral gyrus at the slow‐5 band (0.073‐0.08 Hz). Correlation analysis showed that Clostridium members (*Lachnospiraceae* and *Blautia*) were positively, while *Veillonellaceae* was negatively, correlated with cognition test. Bacteroides was positively correlated with attention and computation, and negatively correlated with the three‐stage command score.

**Conclusions:**

aMCI patients have a specific GM‐intrinsic brain activity‐cognitive function interaction pattern.

## Graphical abstract

Mounting evidences suggest the crosstalk exists between the gut microbiota (GM) and the brain. In this study, amnestic mild cognitive impairment (aMCI) subjects show changes in resting state brain activity and cognitive function that parallel to specific GM bacterial taxal populations, which suggests aMCI patients have a specific interaction pattern of gut microbiota‐intrinsic brain activity‐cognition.

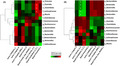

## 1 INTRODUCTION

Alzheimer's disease (AD) is the commonest cause of dementia in the elderly and is clinically characterized by progressive cognitive impairment. Converging evidences from both genetic at‐risk cohorts and clinically normal older individuals suggests that the pathophysiological process of AD begins years before the diagnosis of clinical dementia.^1^, ^2^, ^3^ Both the underlying pathophysiological processes and clinical symptomatology suggest that AD should been considered as a continuum or a spectrum.^4^ Mild cognitive impairment (MCI), recognized as the prodromal stage of AD, refers to a transitional period between normal aging and the dementia stage of this condition.^5^ Among the subtypes of MCI, amnestic MCI (aMCI) carries a substantial risk for progression to AD, with a yearly transition rate of up to 25%.^6^ To date, no effective medical treatment exists to prevent or slow AD progression, and early detection of individuals in the prodromal stage, especially of MCI patients, is of great importance.

Although Aβ peptide accumulation is considered to be a key early event in the pathogenesis of AD, precise pathologic mechanisms remain to be elucidated.^7^ Ongoing work in the field of central nervous system diseases has yielded preliminary evidence of gut microbiota (GM), an important environmental factor, interacting closely with brain functions, including cognition, emotion, and social behavior, suggesting a potential role in AD pathogenesis.^8^, ^9^ Animal studies have shown that intestinal dysbiosis is involved in the initiation, development, and progression of AD, including chronic neuroinflammation, oxidative stress, and neuroimmune‐neuroendocrine pathologies.^8^, ^10^ Recently, an important link has been established between gut‐derived lipopolysaccharide (LPS) translocation to the perinuclear region and the AD‐affected brain.^11^ More recently, Vogt *et al* reported differences in GM of American patients with AD, including decreased Firmicutes, increased Bacteroidetes, and decreased Bifidobacterium.^12^ However, there are few direct data on GM in aMCI patients. Our previous study found that fecal microbial composition of aMCI patients was altered, with increased Bacteroides and decreased several beneficial genus belong to phylum Firmicutes.^13^

Given the difficulty of precisely elucidating human cerebral cellular networks, few studies have established direct links between the GM and cerebral function in vivo.^14^ However, Tillisch et al discovered that intake of fermented milk product probiotics over a four‐week period was associated with significant changes in the intrinsic activity of the resting brain during emotion recognition tasks in healthy female subjects as evaluated using functional magnetic resonance imaging (fMRI).^15^ Furthermore, they grouped the healthy women into two bacterial genus‐based clusters: one cluster with a greater abundance of *Bacteroides* and another with a greater abundance of *Prevotella*. The *Prevotella* group exhibited more negative emotional responses to negative images, and such responses were associated with reduced functional activation of the hippocampus.^16^

On the other hand, because functional alterations often precede structural changes, analysis of intrinsic brain activity is essential for understanding the pathogenesis of aMCI, as well as its early detection. In contrast to task‐based fMRI, resting‐state fMRI (rs‐fMRI) does not require complicated experimental designs that can remove some stimuli or task‐related confounds. It provides a reliable measure of “baseline” brain activity and connectivity. The amplitude of low‐frequency fluctuations (ALFF) technique was found to be reliable and useful in characterizing the intrinsic or spontaneous brain activity during rs‐fMRI evaluation and has been widely used in patients with aMCI or AD.^17^, ^18^, ^19^, ^20^ The low‐frequency range can be usually subdivided into four distinct bands: slow‐5 (0.01‐0.027 Hz), slow‐4 (0.027‐0.073 Hz), slow‐3 (0.073‐0.198 Hz), and slow‐2 (0.198‐0.25 Hz). Slow‐3 and slow‐2 bands mainly reflect white matter signals and high‐frequency physiological noises, respectively, and slow‐4 and slow‐5 bands reflect gray matter signal.^20^ Han et al demonstrated that the widespread abnormalities in intrinsic brain activity of aMCI patients are frequency‐dependent (slow‐4 versus slow‐5).^21^ However, ALFF was also reported to be sensitive to physiological noise. The fractional ALFF (fALFF) approach, relying on filtering, scaling, and normalization to control for physiological or random noise, as well as ventricular contamination and global individual differences, would thus significantly improve sensitivity and specificity in detection of regional spontaneous brain activity.^22^, ^23^

To the best of our knowledge, no study to date has detailed the interactions between intestinal microbiota and brain function as evaluated by fMRI and measures of cognition level in aMCI subjects. Here, we aimed to study how intrinsic cerebral activity (as evaluated by fALFF of rs‐fMRI) and cognitive function associates with the GM profiles in aMCI patients.

## 2 MATERIALS AND METHODS

### 2.1 Participants

This was a cross‐sectional, case‐control study. A total of 42 subjects (aMCI = 20, normal cognition healthy control (HC) = 22) were included. All participants were aged 50 ~ 85 years old, right‐handed, and possessed at least six years of education. Each participant underwent detailed history evaluation, neurological, and neuropsychological assessments including Mini‐Mental State Examination (MMSE)^24^ and the Beijing version of the Montreal Cognitive Assessment (MoCA).^25^

The aMCI participants were recruited consecutively from the Memory Clinic, Department of Neurology at the First Affiliated Hospital, College of Medicine, Zhejiang University. Patients all met criteria for aMCI as described previously by Petersen et al, including memory complaints as verified by an informant, impairment in fulfilling activities of daily living, MMSE scores ≥25, and Clinical Dementia Rating (CDR) scores of 0.5.^26^ HC subjects were recruited in the same proportions of gender and age; most were patient spouses who had lived in the same household for at least 20 years and on the same diet together, while others were recruited from a health‐promotion center in our hospital. All HC subjects had MMSE scores ≥25, CDR scores of 0, and no significant memory complaints.

The exclusion criteria of the participants were as follows: other causes of cognitive impairment; consumed antibiotics, probiotics, prebiotics, or synbiotics within two months prior to fecal sample collection; suffered severe malnutrition, infection, drug or alcohol addictions, irritable bowel syndrome, and inflammatory bowel disease in the last year; with schizophrenia, schizoaffective disorder, or primary affective disorder; with combined severe heart, brain, liver, kidney, lung, and hematopoietic system diseases, and other serious primary diseases; with MR incompatibility; with severe auditory, visual, or motor deficits hampering cognitive testing.

This study was approved by the Ethics Committee of the First Affiliated Hospital, College of Medicine, Zhejiang University. Each participant or their legally authorized caregiver was informed of the purpose of this study, and informed written consent was obtained. See Table 1 for demographic and clinical characteristics of study samples.

**TABLE 1:** Demographic and clinical parameters of the two groups

	HC	aMCI	t/χ^2^	*P* value
n	22	20		
Age (y, means ± SD)	72.7 ± 8.05	68.8 ± 11.2	‐1.33	.191
Sex (Female, %)	13（59.1%）	8（40.0%）	1.53	.217
Spouse (%)	/	15(75%)	/	/
Education (yrs, means ± SD)	10.6 ± 3.32	11.7 ± 3.54	1.07	.296
Smoking (%)	1(4.54%)	1(5%)	0.43	.511
Drinking (%)	2(9%)	1(5%)	7.34	.932
BMI (kg/m^2^, means ± SD)	22.1 ± 2.32	22.8 ± 2.32	0.98	.331
Diabetes (%)	1(4.54%)	2(10%)	0.01	.932
Hypertension (%)	12(54.5%)	8(40.0%)	0.30	.580
Fasting glucose (mmol/l, means ± SD)	5.36 ± 0.74	6.11 ± 1.76	1.83	.074
Hemoglobin (g/l, means ± SD)	140 ± 15.3	145 ± 10.4	1.19	.242
Folic acid (ng/ml, means ± SD)	10.3 ± 5.32	8.84 ± 3.06	1.05	.302
Vitamin B_12_ (pg/ml, means ± SD)	495 ± 270	620 ± 556	0.94	.353
TT4 (nmol/l, means ± SD)	108 ± 23.2	106 ± 25.6	0.24	.812
TT3 (nmol/l, means ± SD)	1.65 ± 0.34	1.62 ± 0.33	0.29	.773
MMSE (means ± SD)	28.3 ± 1.42	27.4 ± 1.66	1.87	.068
MoCA (means ± SD)	26.6 ± 1.74	22.4 ± 2.54*	6.33	.000

Abbreviations: aMCI, amnestic mild cognitive impairment; HC, healthy control; MMSE, Mini‐Mental State Examination; MoCA, Montreal Cognitive Assessment; SD, standard deviation.

* *P* < .05, compared with HC; *t*, the *P* value was obtained by the two‐sample *t* test; *χ* ^2^, the P value was obtained by the chi‐square test.

### 2.2 Clinical data acquisition

Demographic and clinical data including age, sex, years of education, smoking status, drinking status, and comorbidities were obtained by the researcher during subject interview. Each subject's weight and height were measured and the body mass index (BMI) value was calculated. The MMSE was used as a global measurement of cognitive status. The Beijing version of the MoCA is the most widely used version in mainland China and exhibits good internal consistency and general criterion‐related validity. It includes seven cognitive domains (ie, visuospatial/executive function, naming, attention, abstraction, language, delayed memory, and orientation) and is reliably used to differentiate MCI from normal aging and dementia. The CDR scale was used to evaluate dementia severity.^27^

### 2.3 Gut microbiota analyses

Each participant was asked to collect a fresh fecal sample. Stools were preserved at −80°C until analysis. Genomic DNA was extracted from stool samples using a DNA extraction kit (Qiagen, Valencia, CA) according to the manufacturer's protocol, as previously described.^28^, ^29^ The V3‐V4 region of the 16S bacterial ribosomal RNA gene has been targeted by the following primers “forward fusion primers (5’‐CAAGCAGAAGACGGCATACGAGATGTGACTGGAGTTCAGACGTGTGCTCTTCCGATCTBARCODEACTCCTACGGGAGGCAGCAG‐3’) and the reverse fusion primer (5’‐AATGATACGGCGACCACCGAGATCTACACTCTTTCCCTACACGACGCTCTTCCGATCTBARCODEGGACTACHVGGGTWTCTAAT‐3’) (available upon request)” and sequenced using an Illumina® MiSeq platform. All reads were deposited and grouped into operational taxonomic units (OUT) at a sequence identity of 97%. The 16S rRNA reads were processed and compared using Quantitative Insights Into Microbial Ecology (QIIME) version 1.8.0 to calculate α‐diversity, including the Chao 1 and abundance‐based coverage estimators (ACE) indices of species richness, and two widely used diversity indices, the Shannon and Simpson indices of diversity.^13^

### 2.4 Neuroimaging acquisition and analysis

#### 2.4.1 Data acquisition and preprocessing

Functional and structural imaging data were acquired using a 3.0 Tesla MRI scanner (GE Discovery 750 MRI) at the Center for Cognition and Brain Disorders, Hangzhou Normal University, China. Functional images were acquired using an echo‐planar imaging sequence (repetition time/echo time = 2000 ms/30 ms, flip angle = 90°). A total of 43 transverse slices (field of view = 220 × 220 mm^2^, matrix = 64 × 64, slice thickness = 3.2 mm, no interslice gap) aligned along the anterior‐posterior commissure line were acquired. In each scan, 240 volumes were collected and a total scan time of 480 seconds was required. Subjects were instructed to rest with their eyes closed, not to think of anything in particular, and not to fall asleep during scanning. Subsequently, 3D T1‐weighted anatomical images were acquired in the sagittal orientation using a magnetization prepared rapid acquisition gradient‐echo sequence (repetition time/echo time = 8.2 ms/3.2 ms, flip angle = 8°, (field of view = 256 × 256 mm^2^; matrix = 256 × 256; slice thickness = 1 mm; no interslice gap; 176 slices). After each scanning session, the responsiveness of the subjects was evaluated by vocal communication to determine whether they were asleep or awake. Subjects were additionally asked whether they had fallen asleep during the scan.

Images were preprocessed using Resting‐State fMRI Data Analysis Toolkit plus V 1.2 (RESTplus V1.2, www.restfmri.net).^30^ The first 10 functional images obtained from each subject were excluded from analysis. Subsequent images were corrected by slice timing and realignment. Neither translation nor rotation parameters within any given data set in any subject exceeded 2 mm or 2°. Individual 3D T1‐weighted anatomical images were co‐registered to functional images. Using unified segmentation in SPM 12.0, 3D T1‐weighted images were segmented into gray matter, white matter, and cerebrospinal fluid (CSF), and then normalized to Montreal Neurologic Institute (MNI) space. These transformation parameters were then applied to functional images. The normalized data were resliced at a resolution of 3 × 3 × 3 mm^3^ and spatially smoothed with a 6‐mm full width at half maximum Gaussian kernel. Two preprocessing steps were conducted to remove possible sources of variance for each voxel's time course: (1) correction for linear drift and (2) regressing out nuisance variables, including Friston‐24 parameters of head motion (six head motion parameters, six head motion parameters one time point before, and the twelve corresponding squared items), cerebrospinal flow signals, and white matter signals.^31^

#### 2.4.2 fALFF analysis

Fractional amplitude of low‐frequency fluctuation (fALFF) is the ratio of the amplitude in a low‐frequency band to the amplitude in the total frequency band. Respiratory and cardiac fluctuation signals influence data analysis in the 0.073‐0.25 Hz frequency range, and altered functional connections were previously observed only in the slow‐4 and slow‐5 bands.^32^ Here, the typical 0.01‐0.08 Hz low‐frequency range was divided into two sub frequency bands: slow‐4 band (0.027‐0.073 Hz) and slow‐5 band (0.01‐0.027 Hz). For standardization purposes, the fALFF value of each voxel was divided by the mean fALFF within the brain mask, and relevant frequency‐dependent fALFF values were calculated for each subject.

### 2.5 Statistical analyses

Results are presented as numbers with percentages, means with standard deviations (mean ± SD), or medians with interquartile ranges (medians (IQR)) when appropriate. Statistical analyses were performed using the SPSS software package version 16.0 (SPSS Inc, Chicago, IL, USA) and GraphPad Prism 6 (GraphPad Software, Inc). Student's *t* test or Mann‐Whitney test was applied for two‐group comparisons. fALFF values for different bands in the HC and aMCI groups were compared on a voxel‐wise basis using a two‐sample *t* test and mean FD (framewise displacement)^33^ taken as covariates. *P* < .05 corrected with Gaussian random field (GRF) was used. Correlations between fecal GM relative abundance (RA), cognitive assessment results, and fALFF values of the cluster showing significant interactions between groups were performed using Spearman's or Pearson's correlation analysis. Cluster 3.0 (http://bonsai.ims.u‐tokyo.ac.jp/~mdehoon/software/cluster) based on Spearman's or Pearson's correlation coefficient was used to achieve the hierarchical analysis and visualized via the Java TreeView 1.0.5 (http://jtreeview.sourceforge.net/). All statistical tests were two‐sided, and difference achieving values of *P* < .05 were considered statistically significant.

## 3 RESULTS

### 3.1 Demographic and clinical characteristics of enrolled subjects

Demographic and clinical parameters of aMCI and HC groups are summarized in Table 1. The groups did not differ with respect to age, sex, education, BMI, history of smoking, drinking, hypertension, or diabetic status. No differences in standard laboratory findings of fasting glucose, hemoglobin, folic acid, vitamin B_12_, TT3, and TT4 levels were found between the two groups. Cognitive assessment revealed that aMCI and HC groups were matched by MMSE scores; aMCI subjects were found to have lower MoCA scores than HC subjects (*t* = 6.33, *P* < .001). The result of group differences in seven sub‐items of MoCA is shown in Table 2. The aMCI patients had lower score of delayed recall function than HC (*P* = .001). There was no significant difference in the other sub‐items of MoCA score between aMCI and HC groups.

**TABLE 2:** Comparison of subitem MoCA score between the two groups

MoCA subdomains	HC	aMCI	*P* value
	(n = 22)	(n = 20)	
Visuospatial/executive function	5 (4‐5)	4 (3.75‐5)	.631
Naming	3 (3‐3)	3 (3‐3)	.945
Attention	6 (6‐6)	6 (5‐6)	.321
Language	2 (2‐3)	2 (1.25‐2)	.174
Abstraction	2 (1‐2)	1 (0‐2)	.133
Delayed recall	4 (3‐5)	1 (0‐1)*	.001
Orientation	6 (6‐6)	6 (5.25‐6)	.664

Abbreviations: aMCI, amnestic mild cognitive impairment; HC, healthy controls; IQR, interquartile range; MoCA, Montreal Cognitive Assessment.

* *P* < .05 compared with HC group. Data are given as medians (IQR). Group difference was analyzed by Mann‐whitney *U* test method.

### 3.2 Comparison of GM composition between aMCI patients and healthy controls

**FIGURE 1:**
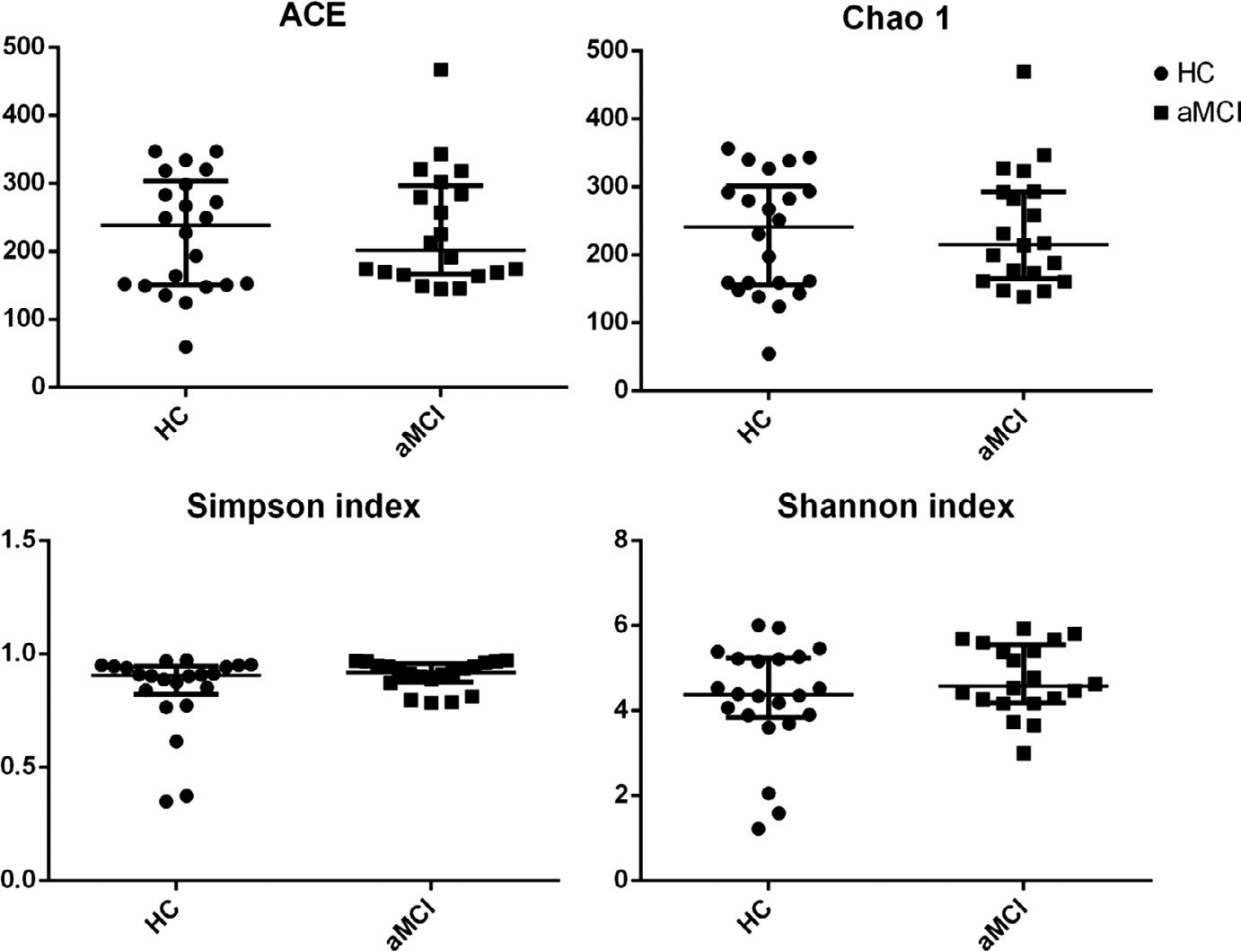
The alpha‐diversity of the GM in aMCI and healthy control subjects depict according to abundance‐based coverage estimator (ACE) and Chao1, Simpson's index, and Shannon's index. Each scatter plot represents the median, interquartile range, minimum, and maximum values. *P* values were determined using a Mann‐Whitney test. HC, healthy control; aMCI, amnestic mild cognitive impairment

To assess differences in fecal microbiota of patients with aMCI compared with those of HC subjects, parallel pyrosequencing was performed. No significant difference in fecal microbiota richness (ACE, Chao 1) or diversity (Simpson's indices, Shannon's indices) among aMCI and HC subjects (Mann‐Whitney; *U* = 210.0, *P* = .801; *U* = 175.0, *P* = .257; *U* = 176.0, *P* = .268) was found (see Figure 1).

**FIGURE 2:**
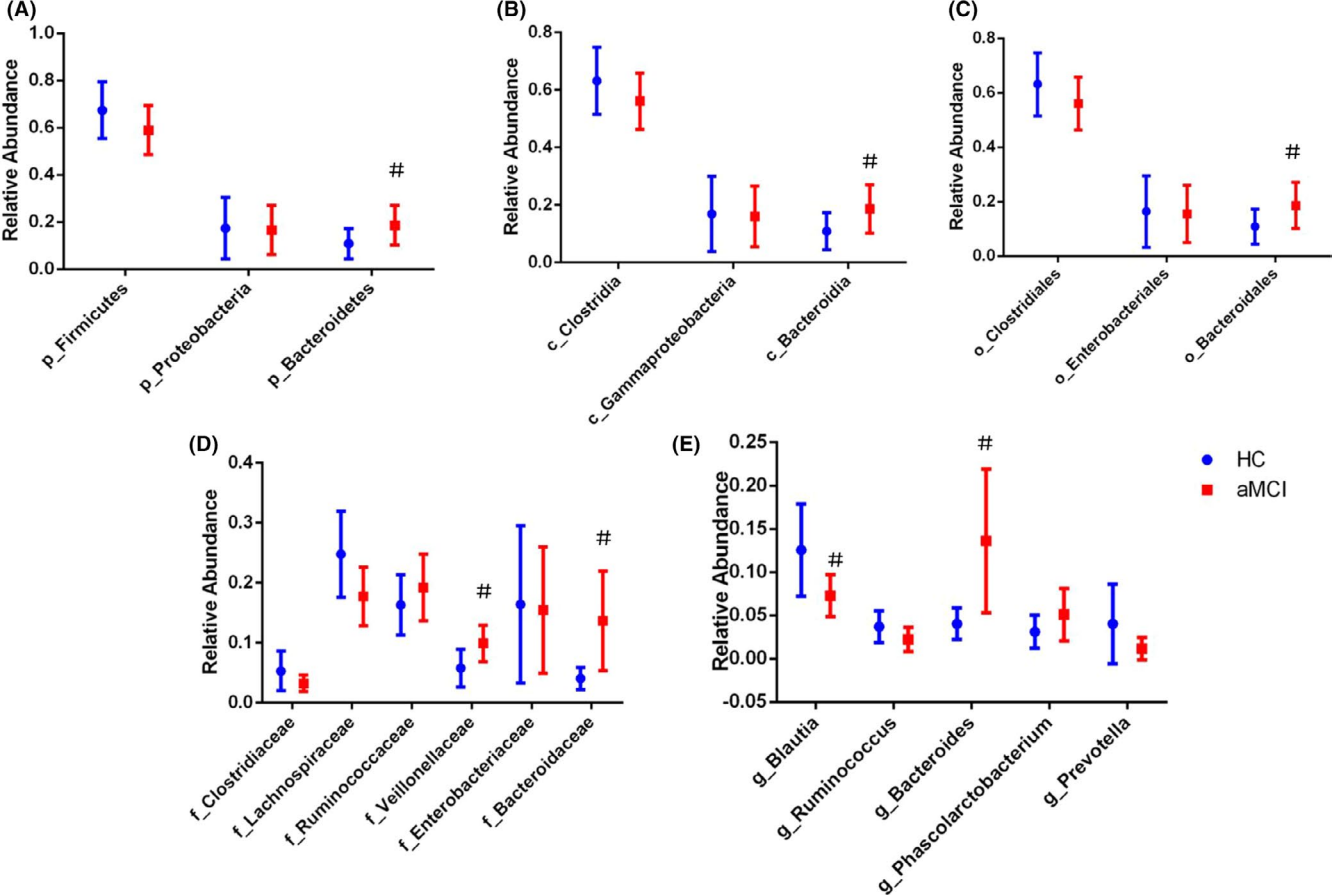
Comparison of the representative taxonomic abundance between aMCI and HC groups (mean value with 95% confidence interval). *t* Test or Kruskal‐Wallis test indicated the significant differences in Bacteroidetes (A) between the two groups, and also in their corresponding class (B), order (C), family (D), and genus (E) (blue, HC; red, aMCI). Note: ^#^ *P* < .05 compared with HC group. Abbreviations: HC, normal cognition healthy control; aMCI, amnestic mild cognitive impairment; p, phylum; c, class; o, order; f, family; g, genus

We subsequently performed taxon‐based analysis, comparing the prevalence of GM at phylum, class, order, family, and genus levels between aMCI and HC groups (see Figure 2). At the phylum level, no significant difference in relative abundance (RA) of Firmicutes and Proteobacteria was found. Bacteroidetes RA was higher in aMCI subjects as compared to those of the HC group (18.64% vs 9.32%, *t* = −2.058, *P* = .045). At class and order levels, aMCI subjects had higher fecal levels of *Bacteroidia* and *Bacteroidales* than HC subjects (18.64% vs 9.32%, *t* = −2.058, *P* = .045) (20.38% vs 9.32% *P* = .004). We additionally matched the prevalence of *Clostridia*, *Gammaproteobacteria*, *Clostridiales,* and *Enterobacteriales* between the two groups. At the family level, *Veillonellaceae* (10.0% vs 5.51%, *P* = .029) and *Bacteroidaceae* (13.63% vs 3.68%, *P* = .023) were more enriched in aMCI than HC subjects. A decreasing trend of *Lachnospiraceae* (17.73% vs 24.31%, *P* = .099) and *Clostridiaceae* (3.22% vs 5.77%, *P* = .088) was noted in aMCI subjects. At the genus level, *Blautia* (7.29% vs 12.91%, *P* = .038) was lower in aMCI subjects while *Bacteroides* was increased in aMCI subjects (13.63% vs 3.68%, *P* = .023). A decreasing trend of *Ruminococcus* (2.23% vs 4.50%, *P* = .075) in aMCI subjects was noted while the RA of *Phascolarctobacterium* and *Prevotella* showed no significant difference among the two groups (*P* = .184 and 0.358, respectively).

### 3.3 fALFF of group differences

**FIGURE 3:**
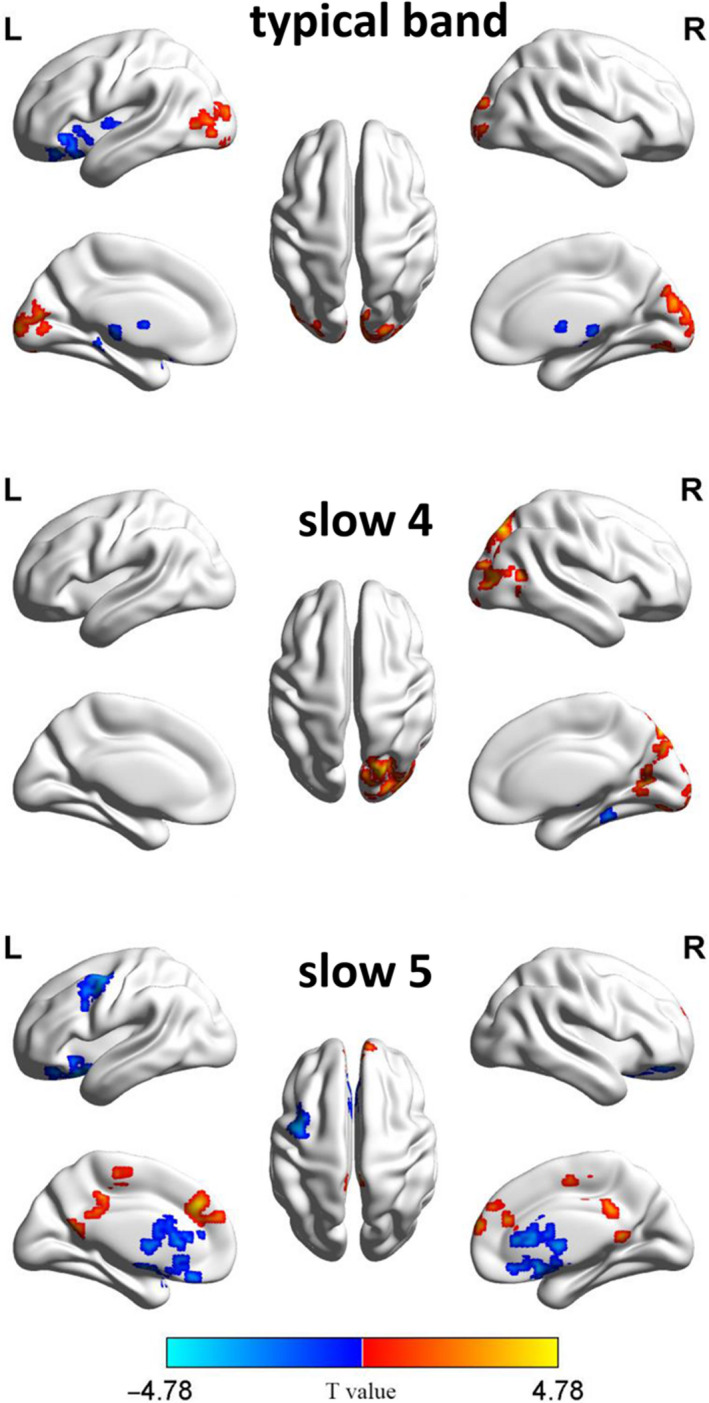
fALFF of group differences in typical band: slow‐4 and slow‐5 bands. Hot color represents higher fALFF in the aMCI than control group in the group, whereas the blue color represents a lower fALFF. The color bar indicated t value

Both increased and decreased regional function was revealed in aMCI subjects relative to controls (Figure 3). Compared to HC subjects, aMCI patients exhibited decreased fALFF values in the cerebellar vermis IV‐V, left lenticular nucleus and pallidum, and increased fALFF values mainly in right lingual gyrus and left middle occipital gyrus at the typical band (0.01‐0.08 Hz). At the slow‐4 (0.027‐0.073 Hz), aMCI patients exhibited decreased fALFF values in the left cerebellum lobules IV‐V; increased fALFF values were noted among aMCI subjects in the right cuneus. At the slow‐5 (0.01‐0.027 Hz), aMCI patients showed increased fALFF values in the left medial superior frontal gyrus and decreased fALFF values in the left precentral gyrus (see Figure 3 and Table 3).

**TABLE 3:** Peak coordinate regions showing fALFF differences in aMCI group compared to HC with MNI coordinates

Brain region (AAL)	Cluster size	Peak MNI coordinates (mm)			BA	T value
		X	Y	Z		
Norm1（0.01‐0.08 Hz）						
Vermis_4_5	342	0	‐54	‐18	/	‐4.4951
Pallidum_L	212	‐12	3	3	/	‐4.7802
Lingual_R	245	21	‐87	‐3	18	4.3368
Occipital_Mid_L	340	‐39	‐75	6	19	4.024
Slow4（0.027‐0.073 Hz）						
Cerebellum_4_5_L	473	‐3	‐48	‐6	30	‐4.3086
Cuneus_R	568	21	‐78	45	7	5.2694
Slow5（0.01‐0.027 Hz）						
Frontal_Sup_Medial_L	167	‐9	36	33	32	4.601
Precentral_L	210	‐48	0	51	6	‐4.366

Note

The resultant T‐maps were thresholded with voxel*P* < .05, cluster *P* < .05 (Gaussian random field theory (GRF) correction for multiple comparisons.

Abbreviations: AAL, anatomical automatic labeling; aMCI, amnestic mild cognitive impairment; BA, Brodmann area; Cerebellum_4_5_L, left cerebellum lobules IV‐V; Cuneus_R, right cuneus; fALFF, fractional amplitude of low‐frequency fluctuations; Frontal_Sup_Medial_L, left medial superior frontal gyrus; HC, healthy control; Lingual_R, right lingual gyrus; MNI, Montreal Neurologic Institute; Occipital_Mid_L, left middle occipital gyrus; Pallidum_L, left lenticular nucleus, pallidum; Precentral_L, left precentral gyrus; Vermis_4_5, vermis IV‐V.

### 3.4 Associations between the cognitive tests, intrinsic brain activities, and gut microbiome

**FIGURE 4:**
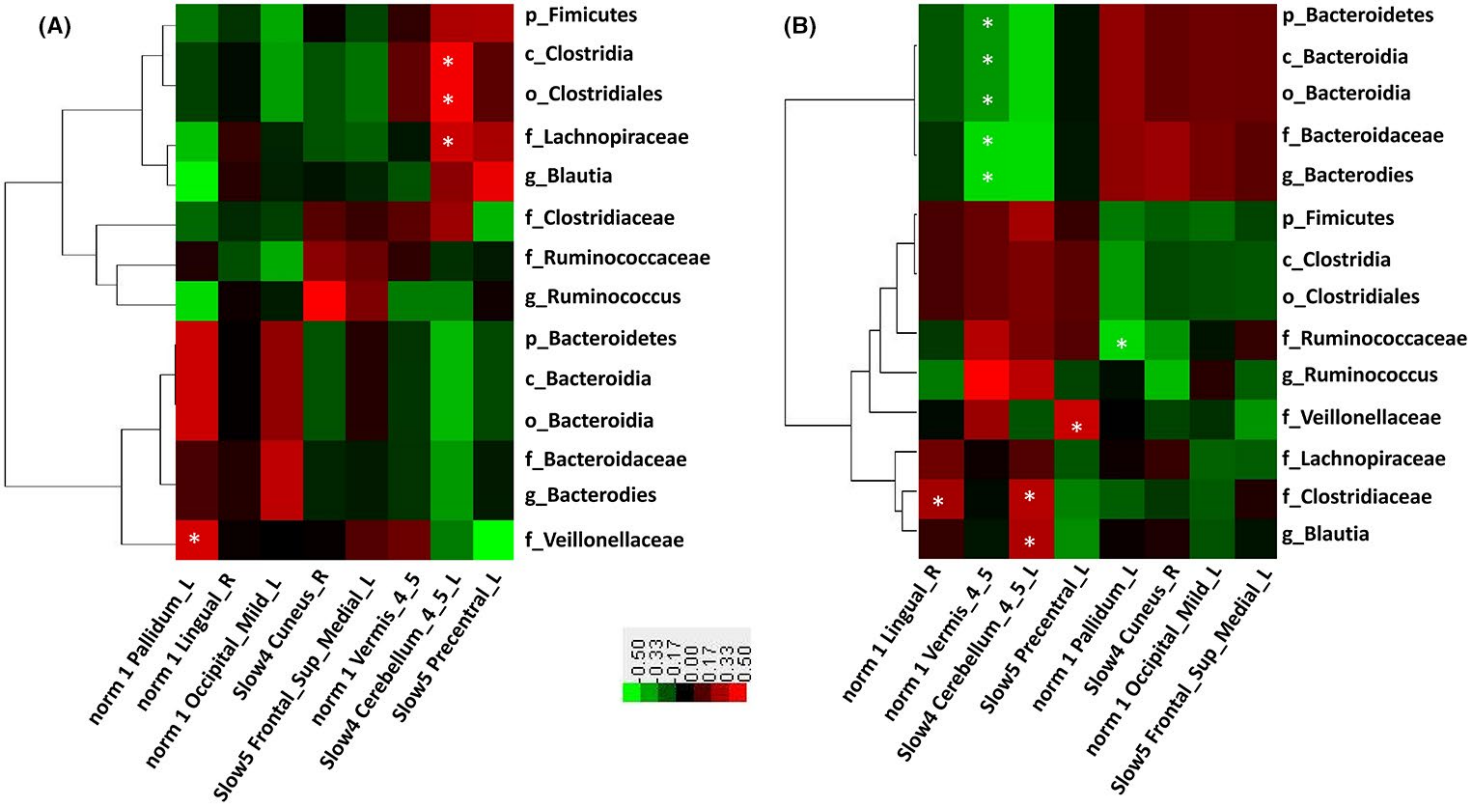
Different gut microbiota and intrinsic brain activity map patterns of HC (A) and aMCI (B) groups. Heatmap showing Pearson's correlation analysis between relative abundance of fecal microbiomes and fALFF value. Red means positive correlation and green means negative; *P < .05

We subsequently explored the potential relationships between alterations in GM relative abundance (RA) and intrinsic brain activities (ie, fALFF values), and cognitive test scores. As shown in Figure 4, aMCI and HC groups had different “GM RA‐intrinsic brain activity interaction” patterns. Specifically, we found that the GM with intergroup differences correlated significantly with fALFF values of several brain regions in both HC and aMCI patients.

In HC subjects, the RA of class *Clostridia* (*r* = .773, *P* = .024), order *Clostridiales* (*r* = .773, *P* = .024), and family *Lachnospiraceae* (*r* = .731, *P* = .039) was found to be positively correlated with fALFF values of left cerebellum lobules IV‐V (Cerebellum_4_5_L) at the slow‐4 band, respectively. The RA of family *Veillonellaceae* (*r* = .771, *P* = .024) was positively correlated with fALFF values of left lenticular nucleus and pallidum (Pallidum_L).

In aMCI patients, the phylum Bacteroidetes (*r* = −.451, *P* = .046), class *Bacteroidia* (*r* = −.451, *P* = .046), order *Bacteroidia* (*r* = −.451, *P* = .046), family *Bacteroidaceae* (*r* = −.567, *P* = .009), and genus *Bacteroides* (*r* = −.567, *P* = .009) were negatively correlated with fALFF values of cerebellar vermis IV‐V. The RA of family *Ruminococcaceae* (*r* = −.498, *P* = .026) was negatively correlated with fALFF values of left lenticular nucleus and pallidum. The RA of family *Veillonellaceae* (*r* = .500, *P* = .025) was positively correlated with fALFF values of left precentral gyrus. The RA of family *Clostridiaceae* was positively correlated with fALFF values of right lingual gyrus (*r* = .516, *P* = .020) at the typical band, and left cerebellum lobules IV‐V (*r* = .458, *P* = .042) at the slow‐4 band. The RA of the genus *Blautia* was positively correlated with the left cerebellum lobules IV‐V (*r* = .466, *P* = .038) at the slow‐4 band.

**FIGURE 5:**
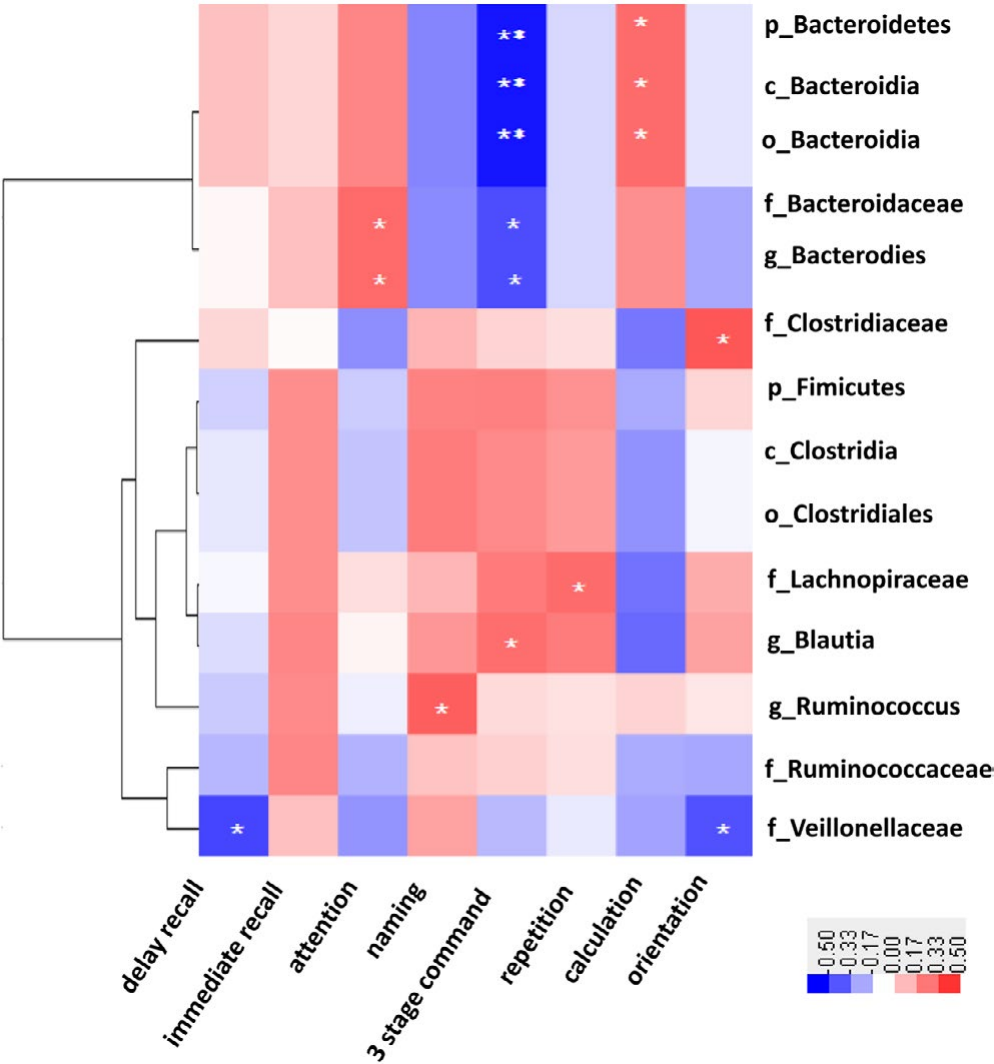
Heatmap showing correlations between fecal microbiome and cognitive test scores of all subjects. Red means positive correlation and blue means negative correlation; *P < .05, **P < .01

Correlation analyses were next performed to evaluate the association between RA of altered microbiomes and cognitive test findings. As shown in Figure 5, the RA of phylum Bacteroidetes, class *Bacteroidia*, order *Bacteroidales*, family *Bacteroidaceae,* and genus *Bacteroides* was positively correlated with scores of attention and calculation (*r* = .364, *P* = .032; *r* = .358, *P* = .037) but negatively associated with three‐stage command test scores (*r* = −.458, *P* = .007). The RA of family *Lachnospiraceae* and genus *Blautia* was positively correlated with repetition and three‐stage command test scores (*r* = .357, *P* = .038; *r* = .352, *P* = .041); the RA of family *Clostridiaceae* was positively correlated with orientation scores (*r* = .410, *P* = .016); the RA of the family *Veillonellaceae,* which also belongs to the phylum Firmicutes, was negatively correlated with orientation and delayed recall scores (*r* = −.340, *P* = .049; *r* = .363, *P* = .035); and the RA of the genus *Ruminococcus* was positively correlated with the naming test scores (*r* = .391, *P* = .022).

## 4 DISCUSSION

Here, we first report cognitive and intrinsic brain activity differences related to intestinal microbial composition in aMCI subjects. In the present study, although there were no significant differences in global diversity or evenness of the complex microbial communities between aMCI and HC subjects, the RA of several gut bacteria in aMCI subjects was distinct from age‐ and sex‐matched control individuals. Moreover, the associations between RA of specific bacterial taxa with the intrinsic activity of the resting brain paralleled changes of corresponding cognition domains.

Firstly, in aMCI subjects, we observed an increase in Bacteroidetes at all levels from phylum to genus. Moreover, the RA of Bacteroidetes was negatively associated with fALFF value of the cerebellar vermis. Recently, mild and moderate AD patients were reported to have an increased RA of Bacteroidetes. The increased Bacteroidetes noted in our aMCI subjects is partially consistent with their results. However, there were various differences between our study and theirs, such as ethnicity, dietary composition, and criteria used to diagnose dementia. Thus, it may be too early to draw any definitive conclusions. Bacteroidetes, a phylum that encompasses a diverse and abundant group of gram‐negative commensal gut bacteria, are thought to be generally beneficial to host health due to their production of polysaccharide metabolites, volatile fatty acids, short‐chain fatty acids (SCFAs), and other nutrients. However, some members of this phylum are opportunistic pathogens, such as Bacteroides fragilis, and the major outer membrane component of these gram‐negative bacteria is lipopolysaccharide (LPS).^34^ When the gastrointestinal tract and blood‐brain barriers alter or increase in permeability due to aging and disease, these bacterial LPS translocate from the gut environment to systemic circulation, triggering systemic inflammation and release of pro‐inflammatory cytokines. Such a chronic inflammatory state is considered to be a key contributor to AD pathology.^34^ Thus, it is necessary to determine the host health state and populations of specific symbionts in order to understand the significance of certain physiological changes in the gut and/or the brain.

The cerebellum has traditionally been considered to be primarily dedicated to motor functions. However, earlier pathologic studies have reported insults in the cerebellum of AD patients.^35^ Clinical and neuroimaging studies have demonstrated that the cerebellum greatly influences thoughts, emotions, as well as language and cognitive processes by functionally connecting and interacting with cerebral networks (ie, dorsal and ventral attention, frontoparietal, default mode, and salience networks).^36^ Indeed, the cerebellum is greatly involved in the pathophysiology of AD. Of note, an insidious decline in the accuracy, speed, and consistency of information processing and cognitive performance in aMCI patients also supports a cerebellar role in the pathophysiology of AD.^37^ Han et al ^21^ also reported a decreased fALFF in the left cerebellum of aMCI subjects. Importantly, this may explain the decreased intrinsic activity of the cerebellum in the aMCI subjects we studied. Meanwhile, in our aMCI patients, the fALFF value of cerebellum vermis IV‐V was negatively associated with RA of phylum Bacteroidetes, class *Bacteroidia*, family *Bacteroidaceae,* and genus *Bacteroides*. It is thus reasonable that a negative relationship exists between Bacteroidetes RA and three‐stage command test scores.

Secondly, several bacterial strains belonging to the class *Clostridia*, phylum Firmicutes, including the families *Lachnospiraceae* and *Clostridiaceae*, genus *Blautia* (of the family *Lachnospiraceae*), and genus *Ruminococcus* were decreased in aMCI subjects. All of the aforementioned bacteria produce SCFAs, which play diverse physiological roles. Furthermore, their numbers correlated positively with cognitive performance, thus representing beneficial effects on hosts. Recently, Sun showed that treatment with *Clostridium butyricum*, a SCFA butyrate producer, could prevent cognitive impairment, Aβ deposits, microglia activation, and production of tumor necrosis factor‐α and interleukin‐1β in the brain of APP/PS1 mice.^38^ There are a number of various acetate and butyrate producers among *Blautia*, and their roles in amelioration of obesity and insulin resistance in rats have previously been reported.^39^ Importantly, *Blautia* spp. were found to correlate with a reduction in mortality caused by acute graft‐versus‐host disease after blood/marrow transplantation due to the anti‐inflammatory effects they exert.^40^ It has been proposed that insulin resistance is related to a higher risk for AD.^41^ Willette et al have reported that, in asymptomatic late middle‐aged individuals at risk for AD, higher insulin resistance predicts temporal and frontal amyloid deposition.^42^ Meanwhile, neuroinflammation plays a significant role in the AD pathogenesis.^43^ As is consistent with our results, preclinical studies have previously confirmed decreased RA of *Ruminococcus* in both APP/PS1 and 5 × FAD mice.^7^ In addition to SCFA production, *Ruminococcus* also modulates gut mucin expression and degradation, which is likely responsible for compromised intestinal permeability in AD.^7^, ^44^, ^45^

Increases in resting‐state activity were found in the left middle occipital gyrus, right lingual gyrus, right cuneus, and left medial superior frontal gyrus in aMCI patients as compared with controls, consistent with previous data.^19^ The first three areas were involved in visuospatial perception and the visual network. And the left medial superior frontal gyrus belongs to prefrontal cortex, which is important for working memory, executive function.^46^ Impairment of the above cognitive function was reported to occur in both aMCI and AD patients. One possible interpretation of this phenomenon is that a compensatory mechanism exists throughout a limited spectrum of along the aging‐MCI‐AD continuum that results in hyperactivation among aMCI patients. These brain regions are activated to compensate for a reduction in function of other brain regions.^18^, ^47^

Here, we found that aMCI and health controls showed different association pattern between intrinsic brain activities, and GM. Bajaj et al demonstrated that elderly populations had a complex interaction between cognition, fMRI activation patterns, and GM abundance. Subjects with both amnestic and non‐amnestic impairment required greater neuronal recruitment of the visuospatial network to achieve the same response compared those with amnestic impairment alone, or unimpaired subjects under task‐based fMRI.^48^ Those findings suggest that a potential mechanism regarding the brain activities may be attributable to the GM, either directly or indirectly. As mentioned above, the metabolites, toxins, and pro‐inflammatory factors from different GM may be a kind of key regulator of the brain activity. Thereafter, longitudinal studies examining the association between intrinsic brain activity and GM metabolites will shed light on this issue.

The results of our study also showed aMCI had altered intrinsic brain activity in specific frequency bands compared with healthy control. Previous studies have demonstrated that pattern of intrinsic brain activity was sensitive to specific frequency bands.^17^, ^21^ And it has been reported that abnormalities of intrinsic brain activity in specific brain regions were frequency‐dependent (particularly, the slow‐4 and slow‐5 bands) both in AD and aMCI patients.^21^, ^49^ Though there are no consistent results, different frequency‐dependent patterns may suggest different oscillations and frequency‐dependent brain spontaneous activities for specific disease state. Further studies combined with electroencephalographic scalp recordings will be helpful to identify the neurophysiological mechanism of the signals located at specific frequency bands.

One limitation of this study is small sample size and preliminary cross‐sectional study. Due to the small study size, the adjustment for multiple comparisons has not been done. The results should be interpreted with caution. Likewise, although stool samples enabled us to detect a wide range of intestinal microflora, it was not possible to differentiate between the luminal and mucosal environment, much less local microenvironments, or regional differences throughout the gut. We did not evaluate bacterial functional changes (ie, metabolic and inflammatory), and our brain scans only studied resting‐state activity. Multi‐modal neuroimaging examinations, including those evaluating brain microstructure and functional connectivity, may reveal interactions between intestinal flora and the brain function more comprehensively.

In summary, we found aMCI subjects have distinct GM compared with age‐ and sex‐matched HC subjects. Changes in resting‐state brain activity and cognitive function run in parallel to specific bacterial taxal populations comprising the GM.

## CONFLICT OF INTEREST

The authors declare no conflict of interest.
